# Mitochondrial *matR *sequences help to resolve deep phylogenetic relationships in rosids

**DOI:** 10.1186/1471-2148-7-217

**Published:** 2007-11-10

**Authors:** Xin-Yu Zhu, Mark W Chase, Yin-Long Qiu, Hong-Zhi Kong, David L Dilcher, Jian-Hua Li, Zhi-Duan Chen

**Affiliations:** 1 State Key Laboratory of Systematic and Evolutionary Botany, Institute of Botany, the Chinese Academy of Sciences, Beijing 100093, China; 2 Graduate University of the Chinese Academy of Sciences, Beijing 100039, China; 3 Jodrell Laboratory, Royal Botanic Gardens, Kew, Richmond, Surrey TW9 3DS, UK; 4 Department of Ecology & Evolutionary Biology, The University Herbarium, University of Michigan, Ann Arbor, MI 48108-1048, USA; 5 Florida Museum of Natural History, University of Florida, Gainesville, FL 32611-7800, USA; 6 Arnold Arboretum of Harvard University, 125 Arborway, Jamaica Plain, MA 02130, USA

## Abstract

**Background:**

Rosids are a major clade in the angiosperms containing 13 orders and about one-third of angiosperm species. Recent molecular analyses recognized two major groups (i.e., fabids with seven orders and malvids with three orders). However, phylogenetic relationships within the two groups and among fabids, malvids, and potentially basal rosids including Geraniales, Myrtales, and Crossosomatales remain to be resolved with more data and a broader taxon sampling. In this study, we obtained DNA sequences of the mitochondrial *matR *gene from 174 species representing 72 families of putative rosids and examined phylogenetic relationships and phylogenetic utility of *matR *in rosids. We also inferred phylogenetic relationships within the "rosid clade" based on a combined data set of 91 taxa and four genes including *matR*, two plastid genes (*rbcL*, *atpB*), and one nuclear gene (18S rDNA).

**Results:**

Comparison of mitochondrial *matR *and two plastid genes (*rbcL *and *atpB*) showed that the synonymous substitution rate in *matR *was approximately four times slower than those of *rbcL *and *atpB*; however, the nonsynonymous substitution rate in *matR *was relatively high, close to its synonymous substitution rate, indicating that the *matR *has experienced a relaxed evolutionary history. Analyses of our *matR *sequences supported the monophyly of malvids and most orders of the rosids. However, fabids did not form a clade; instead, the COM clade of fabids (Celastrales, Oxalidales, Malpighiales, and Huaceae) was sister to malvids. Analyses of the four-gene data set suggested that Geraniales and Myrtales were successively sister to other rosids, and that Crossosomatales were sister to malvids.

**Conclusion:**

Compared to plastid genes such as *rbcL *and *atpB*, slowly evolving *matR *produced less homoplasious but not less informative substitutions. Thus, *matR *appears useful in higher-level angiosperm phylogenetics. Analysis of *matR *alone identified a novel deep relationship within rosids, the grouping of the COM clade of fabids and malvids, which was not resolved by any previous molecular analyses but recently suggested by floral structural features. Our four-gene analysis supported the placements of Geraniales, Myrtales at basal nodes of the rosid clade and placed Crossosomatales as sister to malvids. We also suggest that the core part of rosids should include fabids, malvids and Crossosomatales.

## Background

Rosids [1] comprise one-third of all angiosperm species. Their members are morphologically diverse without apparent universal synapomorphies. Nevertheless, rosids in general have a number of characters that are rare elsewhere in the angiosperms, including nuclear endosperm development, simple perforations in vessel end-walls, diplostemony, mucilaginous epidermis, and epicuticular wax rosettes [2-4]. Recent phylogenetic studies based on both morphology and DNA sequences have demonstrated that subclasses Dilleniidae, Hamamelidae, and Rosidae of Cronquist [5] and Takhtajan [6] are not monophyletic [[1-4,7-15], and references therein]. Some orders, such as Malvales, Salicales, Violales, and Capparales of Dilleniidae and Fagales and Urticales of Hamamelidae have been shown to be rosids, whereas some families of Rosidae, such as Cornaceae, Apiaceae, and Icacinaceae, belong to the asterids [1-4,9-18]. Delimiting the rosid clade and its subclades is therefore central to understanding the phylogeny of eudicots.

Several large-scale phylogenetic analyses of flowering plants at higher taxonomic levels have recently been published based on *rbcL*, *atpB*, 18S rDNA and *matK *sequences, either separately or combined [1-4,9-15]. The results indicated that within the rosid clade there are 12–14 subclades that are well supported and thus recognized as orders. Most rosid orders have been assigned to two large assemblages, fabids (eurosids I) and malvids (eurosids II). Within fabids, there are two subclades, the nitrogen-fixing clade [19] including Cucurbitales, Fagales, Fabales and Rosales, and the COM clade [20] consisting of Celastrales, Oxalidales, and Malpighiales. Nevertheless, inter-ordinal relationships within fabids and malvids, and among fabids, malvids and other rosid orders unassigned to fabids or malvids are either poorly resolved or have low support as measured by jackknife or bootstrap percentages. For example, the placement of Crossosomatales, Myrtales and Geraniales with respect to other rosids still remains uncertain [4]. Recent molecular analyses supported the family Huaceae as sister to Oxalidales in the COM clade [4,21,22], but it is desirable to further corroborate these relationships using a broader taxon sampling. A recent morphological study on supraordinal relationships within rosids [[20], and references therein] produced largely congruent results with DNA-based studies. However, a noteworthy relationship recognized by the morphological data [20] was the grouping of the COM clade of fabids and malvids, which was inconsistent with all previous molecular studies. Therefore, both comprehensive taxonomic sampling and more molecular characters from different genomes are needed to further clarify phylogenetic relationships within rosid clade.

In this study, we present new mitochondrial DNA (mtDNA) sequences, approximately 1,800 base pairs of the mitochondrial gene *matR *from 174 species to re-examine the phylogenetic relationships of rosids within the framework of eudicots [1]. One advantage of mtDNA is the generally observed, reduced level of homoplasy among more distantly related taxa as a consequence of a slow rate of evolution [23-26]; another advantage is that mtDNA sequences belong to different linkage groups from plastid and nuclear genes, and, thus, provide the possibility of combining phylogenetic information from three genomes [27]. Furthermore, this gene has been inherited vertically since it was inserted into *nad1 *group II intron in the common ancestor of non-liverwort land plants [28,29], and no paralogue has been found so far. To date, few large-scale phylogenetic analyses of eudicots or rosids have included sequences from any mitochondrial gene, although their utility has been established in basal angiosperms and some orders and families of angiosperms [27,30-33]. In addition to performing phylogenetic analysis based on *matR *alone, we also analyzed a smaller combined four-gene (*matR*, *rbcL*, *atpB *and 18S rDNA) 91-taxon matrix in an attempt to increase the resolution and internal support. To explore patterns of molecular evolution in *matR *and its contribution to resolving deep phylogenetic relationships, we also conducted a comparative analysis of *matR *and two plastid molecular makers (*rbcL *and *atpB*). The potential effect of RNA-editing in *matR *on phylogeny reconstruction is also evaluated. Our primary objectives are to resolve the deep relationships among orders of rosids and to evaluate the utility of *matR *in large-scale phylogenetic analyses by comparing the results of *matR *with those based on other widely used molecular markers.

## Results

### Sequence variability and evolutionary analyses

**Figure 1:**
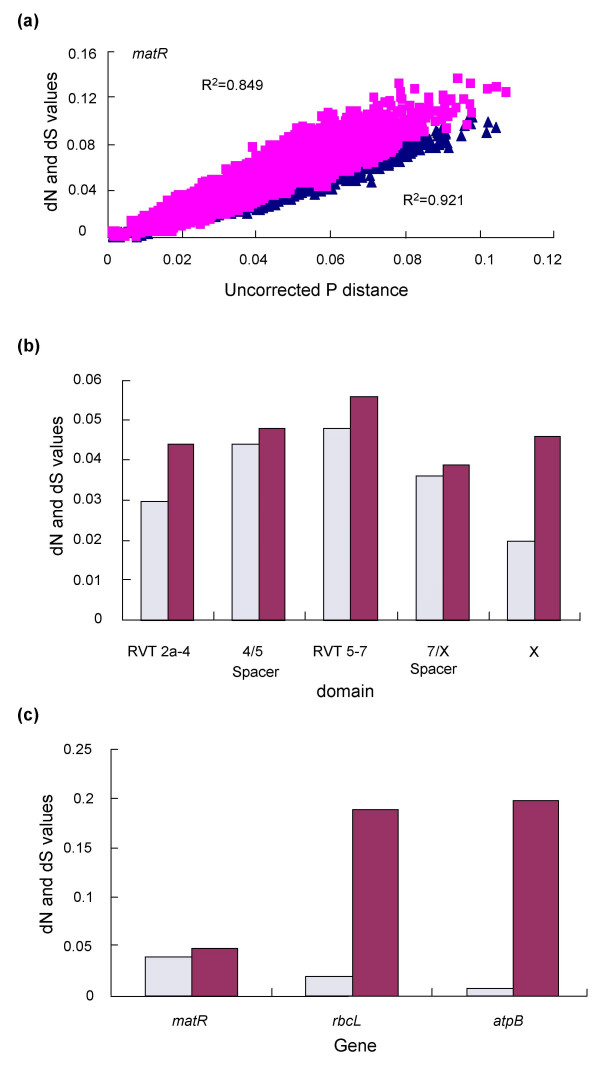
**Evolutionary characteristics of *matR***. (a) Increase of dN (triangles) and dS (squares) values versus the increase of the uncorrected pairwise genetic distance. R^2 ^values show the fit of the relationship to a linear regression model; (b) a comparison of the dN (hatched) and dS (solid) values among different domains of the *matR *gene. The range of domains is determined according to Zimmerly et al [29]; (c) a comparison of the dN (hatched) and dS (solid) values for *matR *and two plastid genes (*rbcL *and *atpB*).

For the 174-taxon matrix of *matR*, nucleotide compositions were not significantly different across the taxa as indicated by a χ^2 ^test (χ^2 ^= 59.804, df = 519, p = 1.0). A relatively high proportion of transversions was found, with an overall transition/transversion ratio of 1.241 under the GTR substitution model (Additional file 2). The overall uncorrected P distance was 0.04, and the largest distance occurred between *Lobelia *and *Hypericum *(11%) and the smallest between *Leea *and *Yua *(0%). Similar rates of change (steps/variable characters) were found among three-codon positions, with 2.56, 2.57 and 2.92 for the first, second, and third codon positions, respectively (Additional file 3). Saturation was not detected for either transitions or transversions at any codon position (data not shown). The selection-pressure plot revealed that both synonymous and nonsynonymous substitution correlate well with uncorrected P distances (Figure 1a), implying that there is no obvious lineage-specific selection pressure within the taxa sampled.

The extent of functional constraints among different domains of the *matR *gene was uneven (Figure 1b); the X domain was the most conserved (dN/dS = 0.43) as found in a previous study [29]. Synonymous substitutions per synonymous site (dS) in the *matR *partition was approximately four times less than those in the plastid partition (*atpB *and *rbcL*) (Figure 1c), showing an extremely low rate of evolution in *matR*, as seen in other mitochondrial regions [23-26]. Nonsynonymous substitutions per nonsynonymous site (dN) in *matR *were near to synonymous substitutions per synonymous site (dS) (dN/dS = 0.81) (Figure 1c), indicating a relaxed evolutionary history of *matR*.

Based on the prediction of the C to U RNA-editing sites in 174 *matR *sequences, none of the sequences were found to belong to processed paralog, which is capable of adversely effecting the phylogeny estimation [34]. A new data matrix, which excluded RNA-editing sites, was constructed on the basis of this prediction. The two data sets yielded nearly identical ML tree topologies except for some weakly supported interior branches (Additional file 8). In addition, we found that the ML tree from the predicted data received less bootstrap support on most branches than that based on original data, indicating that the exclusion of RNA-editing sites reduced phylogenetic signal. Therefore, we directly used genomic sequences for phylogenetic analysis as suggested by Bowe and dePamphilis [34].

### Phylogenetic analysis of *matR*

**Figure 2:**
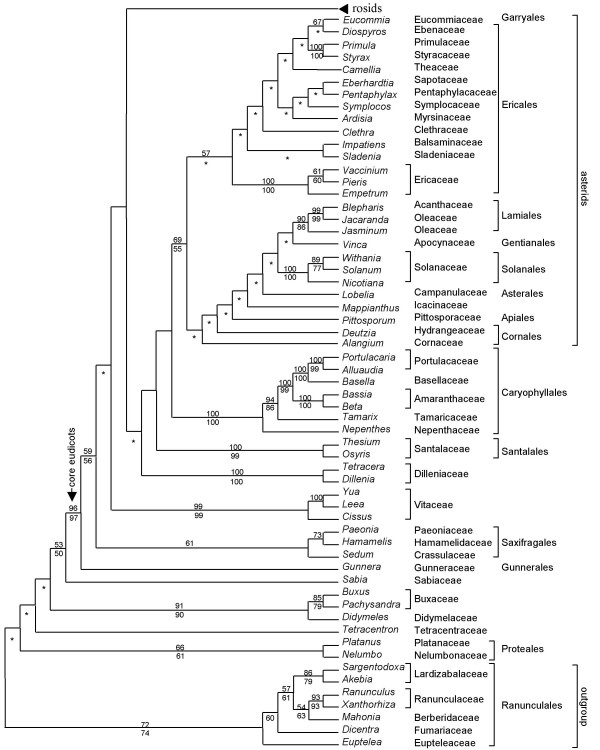
**ML tree (eudicots excluding rosid clade) from the 174-taxon matrix of *matR***. The numbers above branches are ML BS percentages >50; those below are MP BS percentages >50. For nodes where ML and MP analyses differ in topology, only the ML BS percentages are shown; asterisks denote contradictory resolutions between ML tree and MP strict consensus of all shortest trees.

**Figure 3:**
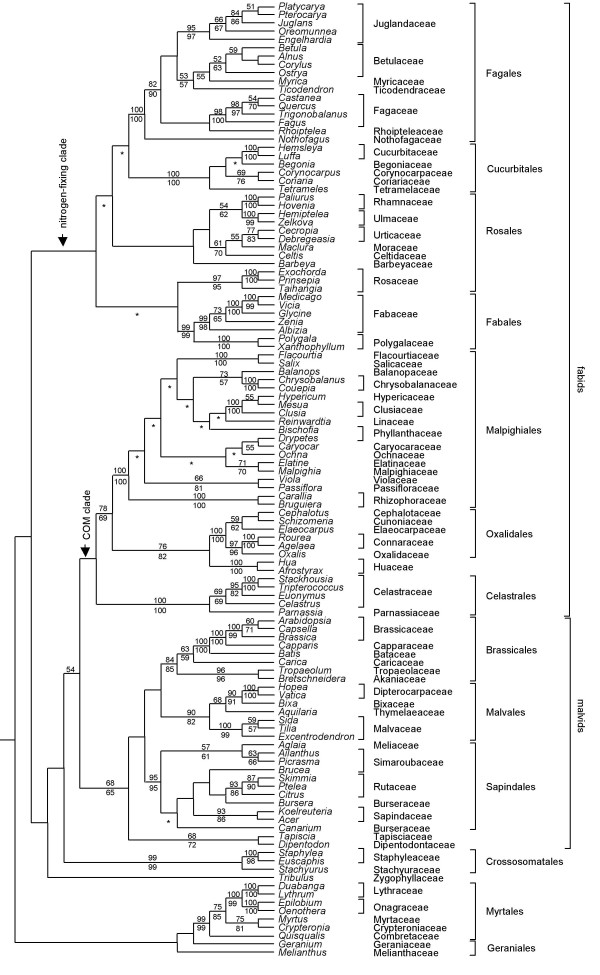
**ML tree (rosid clade) from the 174-taxon matrix of *matR***. The numbers above branches are ML BS percentages >50; those below are MP BS percentages >50. For nodes where ML and MP analyses differ in topology, only the ML BS percentages are shown; asterisks denote contradictory resolutions between ML tree and MP strict consensus of all shortest trees.

Alignment of *matR *sequences resulted in a matrix of 1776 sites, of which 732 (41%) were potentially parsimony-informative. A parsimony analysis generated 34 most-parsimonious trees of 3168 steps with a consistency index (CI) of 0.53 and a retention index (RI) of 0.70. A maximum-likelihood (ML) analysis produced an optimal tree with an lnL score of -23390.64. The ML tree with bootstrap (BS) percentages above each branch and the maximum parsimony (MP) bootstrap (BS) percentages below each branch is presented in Figure 2 and 3. The ML and MP analyses recovered trees with virtually identical topologies; most of differences between ML and MP trees were distributed on extremely short branches. The ML-BS percentages on each of the branches were almost identical with the corresponding MP-BS percentages.

Relationships among the basal eudicots including Proteales, Tetracentraceae, Didymelaceae, Buxaceae, Sabiaceae were not resolved (Figure 2). The core eudicots were strongly supported (96% ML-BS and 97% MP-BS). *Gunnera *(Gunneraceae; Gunnerales) was sister to all other core eudicots (59% ML-BS and 56% MP-BS) as found in a previous study [14]. Relationships among the major core eudicots including rosids, asterids, Caryophyllales, Santalales, Dilleniaceae and Saxifragales were also poorly resolved (Figure 2). The rosid clade was resolved with less than 50% BS.

Within the rosid clade (Figure 3), all orders with multiple representatives formed strongly supported groups except for Rosales and Geraniales. Rosaceae (97% ML-BS and 95% MP-BS) were separated from the remaining members of Rosales, but they were still retained in the nitrogen-fixing subclade of fabids. Fabids did not form a clade in the *matR *tree, and their monophyly [3,12,15] was also rejected by *AU *test (Additional file 4). The COM subclade of fabids was sister to malvids with 54% ML-BS support. *Tribulus*, the single representative of Zygophyllaceae, followed by Crossosomatales, was sister to the above large clade of the COM subclade of fabids plus malvids. Within the COM clade, Huaceae were sister to Oxalidales (76% ML-BS and 82% MP-BS), and alternative topologies without this relationship [3,12] were rejected statistically by the Templeton and *AU *tests (Additional file 4). Malpighiales and Oxalidales/Huaceae were sisters (78% ML-BS and 69% MP-BS), and alternative topologies without this relationship were either rejected or close to the rejection threshold statistically by *AU *test (Additional file 4).

Monophyly of malvids was recovered (68% ML-BS and 65% MP-BS), including Malvales, Sapindales, Brassicales, *Tapiscia *(Tapisciaceae)/*Dipentodon *(Dipentodontaceae) (Figure 3). Brassicales were sister to Malvales with less than 50% BS, and this pair was in turn sister to Sapindales with less than 50% BS. *Dipentodon *plus *Tapiscia *(68% ML-BS and 72% MP-BS) were sister to all other malvids.

### Combined analysis

**Figure 4:**
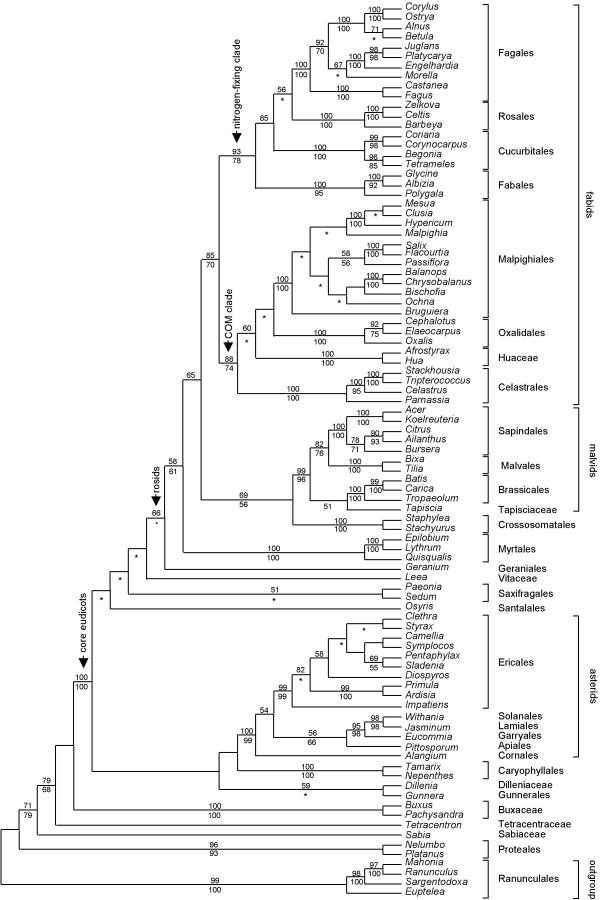
**ML tree from the combined four-gene matrix of *matR*, *rbcL*, *atpB *and 18S rDNA**. The numbers above branches are ML BS percentages >50, and those below are MP BS percentages >50. For nodes where ML and MP analyses differ in topology, only the ML BS percentages are shown; asterisks denote contradictory resolutions between ML tree and MP strict consensus of all shortest trees.

The four-gene matrix consisted of 6197 characters, of which 1637 (26%) were potentially parsimony-informative. A parsimony analysis produced 25 most parsimonious trees of 10591 steps with a CI of 0.36 and a RI of 0.49. ML analysis generated an optimal tree with an lnL score of -65288.16. The maximum likelihood (ML) tree with BS percentages above each branch and the maximum parsimony (MP) BS percentages below each branch is presented in Figure 4. Data partitions and tree statistics for all analyses are presented in Table 1. Comparison of supported supraordinal nodes within rosids is presented in Table 2. The topology of the ML-based analysis was virtually identical with that of the MP-based analysis. The ML-BS percentages were almost identical with those of the MP-analysis as in the analysis of the *matR *alone.

**Table 1:** Data partitions and tree Statistics for each of the analyses. Data for *matK *are from reference [15].

Data partition	Character	CI	RI	Variable Character	% variable character	Pi	% Pi	Steps	*Rate of change
*matR*	1770	0.61	0.65	996	0.56	508	0.29	2160	2.17
*rbcL*	1395	0.28	0.46	595	0.43	448	0.32	3567	5.99
*atpB*	1371	0.31	0.49	600	0.44	449	0.33	3124	5.21
18S rDNA	1661	0.36	0.50	418	0.25	236	0.14	1545	3.70
*matK*	1749	0.14	0.63	1221	0.70	1083	0.62	20801	17.03
*rbcL*-*atpB*	2766	0.29	0.47	1231		897	0.32	6760	
*rbcL*-*atpB*-*matR*	4536	0.36	0.50	2227		1405	0.31	8998	
*rbcL*-*atpB*-18S	4427	0.30	0.47	1648		1132	0.26	8370	
*rbcL*-*atpB*-18S-*matR*	6197	0.36	0.49	2644		1640	0.26	10604	

Pi, parsimony informative; CI, consistency index; RI, retention index.

* Steps/variable characters [12].

**Table 2:** Comparison of the ML-BS percentages for supraordinal nodes within rosids in each of the analyses.

Node	*maatR*	*rbcL*	*atpB*	18S	*matK*	*rbcL*/*atpB*	*rbcL*/*atpB*/*matR*	*rbcL*/*atpB*/18S	*rbcL*/*atpB*/18S/*matR*
Rosids (not including Vitaceae)	<50	nr	<50	nr	**95**	<50	**72**	<50	**66**
Geraniales/remaining rosids	nr	<50	nr	nr	nr	<50	nr	<50	**55**
Crossosomatales/malvids	nr	nr	<50	nr	nr	**64**	**75**	<50	**61**
fabids	nr	**51**	nr	nr	**52**	**62**	**83**	**68**	**85**
Nitrogen-fixing clade	<50	<50	<50	nr	<50	**74**	**99**	**68**	**92**
Fagales/Rosales	nr	<50	nr	nr	nr	**56**	**53**	**51**	**58**
Fagales/Rosales/Cucurbitales	**55**	<50	nr	nr	nr	nr	**69**	**57**	**85**
COM clade	**52**	<50	nr	nr	**60**	<50	**88**	<50	**88**
Oxalidales/Huaceae	**76**	nr	nr	nr	--	nr	nr	nr	nr
Malpighiales/Oxalidales	**82**	nr	nr	<50	nr	<50	**63**	<50	**59**
malvids	**68**	**50**	<50	nr	nr	**90**	**100**	**87**	**99**
Sapindales/Malvales	<50	**69**	nr	nr	nr	**81**	**83**	**84**	**82**
Brassicales/Malvales	nr	nr	<50	nr	**89**	nr	nr	nr	nr

The node name is listed when it is resolved with >50% support (boldface) in any of these analyses. "nr" (not resolved) denotes unresolved node, whereas "--" refers to taxa/clade that not sampled. Data for *matK *are obtained from the MP-JK support in reference [15].

The topology of the four-gene analysis was largely congruent with that resulted from the analysis of *matR *alone (Figure 2 and 3), but with higher bootstrap percentages, especially on deeper nodes. The core eudicots were strongly supported (100% ML and MP BS). The rosid clade (excluding Vitaceae) was resolved with 66% BS support in the ML tree. Within rosids, *Geranium *was resolved as sister to a clade including all other rosid members (58% ML-BS and 61% MP-BS) in the ML tree, whereas the genus was excluded from rosids and nested within Saxifagales in the MP strict consensus tree. Myrtales (100% ML and MP BS) were sister to a combined clade (65% ML-BS) of fabids/malvids plus Crossosomatales. Crossosomatales (100% ML and MP BS) were sister to well-supported malvids with 69% ML-BS and 56% MP-BS support.

Monophyly of fabids was recovered (85% ML-BS and 70% MP-BS), and the sister relationship of the COM subclade of fabids with malvids found in the analysis of *matR *alone was rejected by all statistical tests (Additional file 4). All orders within fabids were monophyletic, including Oxalidales (100% ML and MP BS), Malpighiales (100% ML and MP BS), Celastrales (100% ML and MP BS), Fabales (100% ML-BS and 95% MP-BS), Fagales (100% ML and MP BS), Rosales (100% ML and MP BS), and Cucurbitales (100% ML and MP BS). Despite the typically high support of these orders, relationships among them were relatively weakly supported. There were two large subclades in fabids; one is the nitrogen-fixing clade with 93% ML-BS and 78% MP-BS support, and the other is the COM clade with 88% ML-BS and 74% MP-BS support (Figure 4). Huaceae were grouped with Oxalidales/Malpighiales with 60% BS support in ML tree, but alternative topologies without this relationship [3,12] were not rejected statistically.

Monophyly of malvids was strongly supported (99% ML-BS and 96% MP-BS); they consisted of Malvales (100% ML and MP BS), Sapindales (100% ML and MP BS), Brassicales (100% ML and MP BS), and *Tapiscia *(Tapisciaceae). Malvales were sister to Sapindales with 82% ML-BS and 76% MP-BS support, but alternative topologies without this relationship [12,15] were not rejected statistically. *Tapiscia *(Tapisciaceae) was resolved as sister to Brassicales with <50% ML-BS and 51% MP-BS support.

## Discussion

### Phylogenetic relationships and their robustness

Both bootstrap and jackknife percentages have generally been considered as good indicators of the robustness of clades in phylogenetic trees. However, short internal branches, likely the result of rapid radiations that occurred during earlier periods of flowering plant evolution [4,35], make phylogenetic reconstruction less accurate [36-38]. We noticed that, in our case, ML analyses resolved more inter-ordinal relationships with greater internal support than those with MP (Figure 2, 3 and 4), and most such cases involve clades with short internal branches (Additional file 6 and 7). In addition, most cases of contradictory resolution between ML and MP trees occur on those extremely short internal branches (Additional file 6 and 7). Several simulation studies have shown that model-based methods outperform parsimony in reconstructing short branches located deep in the tree if saturation does not occur [39-41]. Therefore, our discussion will be based on the ML tree although in general terms the two methods produced highly similar estimates of overall relationships and support.

The topology of the *matR *tree shows similar relationships among major eudicot lineages as those based on plastid genes *rbcL*, *atpB *and *matK *in previous separate or combined analyses [12-15]. Clades occurring at basal nodes include Proteales, Trochodendraceae, Buxaceae, and Sabiaceae. Core eudicots are strongly supported and consist of Gunnerales, Dilleniaceae, Caryophyllales, Santalales, Saxifragales, rosids, and asterids. The four-gene data set did not resolve relationships among major eudicot clades, including the rosids, asterids, caryophyllids, Santalales, and Saxifragales. Most rosid orders are well supported in both *matR *and four-gene trees. These orders, including their composition and phylogenetics have been discussed previously [4,42]. Here we mainly focus on higher-level relationships that are different and compare them with other recent studies. Some clades do not receive strong support, but they nevertheless warrant attention in future studies.

#### Rosids

The rosid clade (excluding Vitaceae) has been recovered with low to high bootstrap support in recent phylogenetic analyses of the angiosperms [3,12,15,43,44]. Low support for rosid clade was obtained in our four-gene analysis, and relatively short internal branch lengths were observed for the rosid node in both the *matR *and the four-gene trees (Additional file 6 and 7). Likewise, when we examine support for the rosid clade from the four single-gene matrices as well as various combinations of them we found that this clade was either not present or showed only low ML-BS support (Table 2), which is similar to some earlier studies [10,12,13]. Like three-gene analysis [3] and those of nearly complete plastid genomes [43,44], our four-gene analysis also showed that Vitaceae are sister to rosids, but received less than 50% ML-BS support.

#### Geraniales, Myrtales and Crossosomatales

Previous analyses have produced several positions for the representatives of these three orders but they have never received more than 50% JK or BS support. Therefore, they are still among the major higher-level questions within the rosids [4]. In this study, analysis of *matR *alone did not resolve their placements with greater than 50% bootstrap support, but the four-gene analysis did. In addition, it is also worth noting that Crossosomatales were resolved as a sister to a larger clade, including the COM subclade of fabids and malvids, with slightly less than 50% bootstrap support in the analysis of *matR *alone (results not shown). There are two morphological characters supporting the position obtained for Crossosomatales in this analysis: (1) arillate seeds are conspicuous in the COM clade of fabids, and they are also present in malvids and Crossosomatales although less prominent in the last two clades [20]; (2) free carpels in which the upper part is postgenitally united at anthesis, which appear to be restricted to Malvales and Sapindales of malvids, some Crossosomatales, and Saxifragales [20,45,46]. Therefore, we suggest that Crossosomatales may belong to malvids or a larger clade including the COM subclade of fabids and malvids.

#### Fabids

This large clade includes Malpighiales, Oxalidales, Zygophyllaceae, Celastrales, Cucurbitales, Fagales, Fabales, and Rosales. Our four-gene analysis recovered this clade with moderate BS support, similar to the three-gene analysis of Soltis et al. [3]. However, our analysis of *matR *alone did not recover fabids as a clade, and their monophyly is also rejected by the *AU *test. Instead, an additional sister relationship between the COM subclade of fabids and malvids was recognized, albeit with low ML-BS support. This conflicting resolution may arise from a different history or evolutionary phenomena for *matR *than the other partitions. Support for fabids primarily comes from the two plastid (*rbcL *and *atpB*) and nuclear genes (18S rDNA; Table 2), although addition of *matR *improved resolution within fabids. We note that a sister relationship of the COM subclade of fabids and malvids was moderately supported by floral structural features, but there was only weak support for the fabids from reproductive features [20], particularly an inner integument that is thicker than the outer at the time of fertilization. Other supporting characters [20] include: (1) contorted petals, (2) a tendency towards polystemony, (3) a tendency towards polycarpelly, and (4) integuments often free from each other and from the nucellus; none of these are particularly robust (most are tendencies). Thus, the deepest split within rosids might be between the nitrogen-fixing clade and a large clade including malvids, the COM subclade of fabids, Crossosomatales and Zygophyllaceae (Figure 3), as suggested by Endress and Matthews [20], not between fabids and malvids. It is obvious that more molecular data from all three genomes will be required to further assess whether this novel relationship is locus-specific or general. Our four-gene analysis also identified a larger assemblage of orders with low BS support including fabids, malvids and Crossosomatales, which constitutes the core part of rosids.

There are two major subclades within fabids, the nitrogen-fixing clade [19] and the COM clade [20]. Our four-gene analysis is basically in agreement with those based on three genes [3] but obtains higher support for these two subclades. Within the nitrogen-fixing clade, the sister relationship of Cucurbitales and Fagales was supported in various analyses [3,47]; however, our four-gene analysis does not recognize their sister relationship. In contrast, the sister relationship of Fagales and Rosales was weakly supported in the ML tree, and then they grouped with Cucurbitales to form a larger clade with moderate ML-BS support. These three orders each contain actinorhizal plants with roots nodulated by strains of *Frankia *[48]. Previous molecular analyses have recognized these actinorhizal plants as a clade [47,49], but the taxonomic sampling in these analyses seems to be inadequate for evaluating their relationships. Our results support the hypothesis that the actinorhizal plants originated separately from Fabaceae and Ulmaceae, which are nodulated by rhizobial bacteria [4,19].

In the COM clade, Celastrales have been resolved as sister to Oxalidales in previous studies [9,15,31]. In a more recent multi-gene analysis, Celastrales were recognized as sister to Malpighiales with high JK support [21], consistent with the result of Chase et al. [9]. In our analysis of the *matR *alone, Malpighiales and Oxalidales appeared as sister groups, consistent with several previous analyses [3,12,14], but with apparently higher support; in our four-gene ML tree, they were also resolved as sister groups, but with a decreasing BS support, indicating this signal is primarily derived from the *matR *gene (Table 2); alternatively, the weaker support could be the result of sparser sampling in the four-gene analysis. Analysis of the *matR *matrix placed Huaceae as sister to Oxalidales with moderate support, in agreement with other recent results [4,21,22], whereas our four-gene analysis demonstrates different resolutions between MP and ML trees: the MP analysis resolves Huaceae as sister to Celastrales with <50% BS support, whereas the ML analysis recognizes Huaceae as sister to Oxalidales plus Malpighiales with low BS support.

Malpighiales are a large order including more than 30 families [1], and they have received strong support in previous analyses [3,12,15]. Some families of Dilleniidae sensu Cronquist [5], such as Ochnaceae, Clusiaceae, Violaceae, Passifloraceae, Salicaceae and Flacourtiaceae are included in Malpighiales. In the *matR *tree, Salicaceae s.l. (including some former Flacourtiaceae [50]) form a strongly supported clade (BS 100%); *Caryocar *of Caryocaraceae and *Drypetes *of Putranjivaceae form a weakly supported clade (55% MP-BS). *Balanops*, the only genus of Balanopaceae, was previously supposed to be related to Fagales because of similar pollen and a cupule-like structure [5]. The *matR *analyses support a position of Balanopaceae in Malpighiales, in agreement with the results of the three-gene analysis [3] and the recent morphology-based study [51].

#### Malvids

Both *matR *alone and the four-gene combined analyses resolve malvids as a monophyletic clade, as has been found in other analyses [3,12,15,30]. In our analysis of *matR *alone, *Dipentodon *(Dipentodontaceae), with uncertain position in APG (2003) [1], was resolved as sister to *Tapiscia *(Tapisciaceae) with low support, which is consistent with another recent analysis [30]. Our analysis of *matR *alone did not resolve relationships of Malvales, Brassicales and Sapindales with greater than 50% BS support, but in our four-gene analysis, the sister-group relationship of Malvales and Sapindales received a moderate BS support, in agreement with the result (51% MP-JK) of three-gene analysis of Soltis et al. [3] and the result (89% MP-BS) of four-gene analysis of Nickrent et al. [31]. Malvales and Sapindales share two morphological characters, i.e., "a tendency towards the presence of several (more than two) meiocytes in an ovule and elaborate apocarpy" [20].

### Potential of *matR *in large-scale phylogenetic studies

Our analysis of *matR *alone produced a tree highly congruent with previous studies of single and multiple genes [3,12,15]. In particular, the main contribution of the *matR *data appears to be for estimating support of orders. When supraordinal relationships within the rosid clade are compared on the basis of individual genes, *matR *data resolves more nodes with ML-BS support >50% than *rbcL*, *atpB *or 18S rDNA (length corrected) and is similar to *matK *alone and *rbcL*-*atpB *combined (Table 2). In addition, when *matR *is combined with *rbcL*-*atpB *or *rbcL*-*atpB*-18S rDNA data, additional supraordinal relationships with BS support >50% occur (Table 2). This indicates that mitochondrial *matR *is suitable for reconstructing angiosperm phylogeny at higher levels.

The *matR *gene exhibits two outstanding evolutionary features, a slow rate of evolution and relaxed selection (Figure 1c). For phylogenetic analyses in general, genes that evolve relatively slowly are likely to contain fewer homoplasious substitutions, but then are also expected to have fewer informative sites. Obviously, slowly evolving *matR *should provide less phylogenetic information than plastid genes like *rbcL *and *atpB*, and this should affect its resolving power on short internal branches due to the reduction of phylogenetic signal [36,52]. However, this reduction is at least partially offset by relaxed evolutionary constraints, which leads to more nonsynonymous substitution sites at otherwise conservative first and second codon positions. As a result, the *matR *data has more variable characters and parsimony-informative sites (Pi) compared to the other three genes (length corrected) (Table 1). Although both *matR *and plastid *matK *have experienced a relaxed evolutionary history [15], *matR *(Table 1) provides a significantly higher consistency index (CI) and slightly higher retention index (RI) than significantly more rapidly evolving *matK *[[15], and references therein].

## Conclusion

Analyses of matR sequences alone or combined with atpB, rbcL, and 18S rDNA have provided new insights into several deep relationships among rosid lineages, albeit with low support, including the grouping of malvids and COM subclade of fabids from single matR gene analysis, and the placements of Geraniales, Myrtales and Crossosomatales from the combined four-gene analysis. At ordinal and deeper nodes, *matR *provides many informative sites with less homoplasy, which makes it suitable in higher-level angiosperm phylogenetics. Mitochondrial matR sequences have produced a different topology when combined with plastid and nuclear sequences, and therefore, more genes from the mitochondrial genome should be used in combination with plastid and nuclear genes to further investigate the results presented here, although there are major problems to be overcome with transfers of some gene to the nuclear genome and unusual patterns of molecular evolution for some mitochondrial genes, such as atp1 and coxI, used in monocot phylogenetics [53].

## Methods

### Taxon sampling

For this study, a total of 174 *matR *sequences representing 118 families of eudicots and 72 families of rosids, with representatives from 59% of fabid families and 41% of malvid families [1] were included. Of them, 93 matR sequences were newly generated. Vouchers are deposited in either the herbarium of the Institute of Botany, Chinese Academy of Sciences, Beijing, People's Republic of China (PE), or the Herbarium, Royal Botanic Gardens, Kew, UK (K). In addition to the 174-taxon *matR *matrix, we also analyzed a smaller four-gene combined matrix by combining the *matR *sequences with previously published sequences of *rbcL*, *atpB*, and 18S rDNA available from GenBank. The combined dataset consisted of 91 taxa. When possible, the same species was used for all four genes. The taxa and collection information have been listed in Additional file 1

### DNA extraction and sequencing

For each of the 93 specimens newly sequenced for matR, fresh leaves were frozen or dried in silica gel [54]. Total genomic DNAs were isolated following procedures described in [55]. The primers matR 26F (5' GACCGCTNACAGTAGTTCT 3') and matR 1858R (5' TGCTTGTGGGCYRGGGTGAA 3') were used for both PCR amplification and sequencing. Two additional internal primers, matR 879F (5' ACTAGTTATCAGGTCAGAGA 3') and matR 1002R (5' CACCCACGATTCCCAGTAGT 3'), were also used in sequencing. These internal primers are not universal for all sampled taxa, and therefore, two additional sequencing primers were designed, matR-F3 (5' GGACACACCTGCGCGGATTA 3') and matR-R3 (5' ATCTAGGATAGGCRGCCAACC 3').

PCR was performed using a Perkin Elmer 9600 thermocycler (Norwalk, Connecticut, USA). PCR products were purified using Wizard PCR purification (Promega, Madison, Wisconsin, USA). Sequencing reactions were performed using the PRISM Dye Terminator Cycle Sequencing Ready Reaction Kit (Applied Biosystems, Inc., ABI, Foster City, California, USA), and the products were analyzed using an ABI 377 DNA sequencer, all following the manufacturer's protocols.

### Alignment and Data matrix

The174 *matR *sequences were first aligned at the amino acid level using Clustal X [56], and then the corresponding DNA sequence alignment was constructed according to the protein sequence alignment using PAL2NAL program [57], followed by some manual adjustment. The smaller combined data matrix with 91 taxa was constructed by combining newly generated *matR *sequences with sequences of the three other genes from GenBank. The three protein-coding genes (*matR*, *rbcL *and *atpB*) used in combined matrix were aligned independently with the same procedure as described above. For 18S rDNA, some ambiguous regions were excluded because positional homology could not be established; a total of 61 ambiguously aligned positions were excluded. Autapomorphic insertions and ends of sequences were removed from each alignment. Alignments are available on TreeBASE [58] under M3533 and M3534.

### Phylogenetic analyses

The 174-taxon *matR *matrix and the four-gene combined matrix with 91 taxa were analyzed with maximum parsimony (MP) and maximum likelihood (ML) methods. Ranunculales were designed as outgroup based on topologies of the eudicots in previous large-scale angiosperm studies [3,9,12,13,59]. Equally weighted MP analysis was performed in PAUP* v4.0b10 [60] using 1,000 random replicates of tree-bisection-reconnection (TBR) heuristic searches with a maximum of 1,000 trees held per TBR search. Robustness of clades under MP analysis was evaluated by non-parametric bootstrap using 500 pseudo-replicates with 100 random additions per replicate. For ML analyses, the optimal model and parameters were determined using the hierarchical likelihood ratio tests (hLRTs) as implemented in Modeltest v.3.6 [61], and analyses were implemented in PHYML v.2.4.4 [62] under GTR+Γ model for 174-taxon *matR *matrix and GTR+I+Γ for four-gene combined matrix with all parameters for each data matrix (Additional file 2). Support was estimated by non-parametric bootstrap using 1000 replicates. We used the following descriptions and ranges in the text for describing bootstrap (BS) support in ML and MP analysis: low, up to 75%; moderate, 76–85%; high, 86–100% [63].

Several potential data partitions in the combined matrix were analyzed to compare their phylogenetic signal and contribution to results. These data partitions include each of the four genes, plastid genes (*rbcL*-*atpB*), plastid plus mitochondrial gene (*rbcL*-*atpB*-*matR*), plastid plus nuclear genes (*rbcL*-*atpB*-18S), and plastid plus mitochondrial plus nuclear genes (*rbcL*-*atpB*-18S-*matR*). The optimal models and parameters were derived from each partition (Additional file 2). In addition, analyses based on the three-codon positions in *matR *were also conducted on 174-taxon *matR *matrix to compare variation and phylogenetic signal.

To assess alternative phylogenetic hypotheses, we employed the Templeton [64] and winning-site [65] tests as implemented in PAUP* v4.0b10 under MP, and the Shimodaira-Hasegawa (SH) [66] and approximately unbiased (AU) [67] tests under ML as implemented in CONSEL [68]. Constraint trees of alternative topologies were generated using MacClade v4.06 [69]Additional file 5.

### Sequence variability and pattern of molecular evolution

We used PAUP* v4.0b10 [60] to analyze homogeneity of nucleotide composition, transition/transversion ratios and saturation. PAML v3.15 [70] and MEGA v 3.1 [71] were used to calculate synonymous substitutions per synonymous site (dS) and nonsynonymous substitutions per nonsynonymous site (dN) for each gene. We compared the dS and dN values among three protein-coding genes (*matR*, *rbcL *and *atpB*) to test for differences in rates and constraints. Such estimation was also performed for different domains in *matR *to evaluate the distribution of the variation. We plotted uncorrected pairwise sequence divergence distances against corresponding dS and dN values to test change in lineage-specific selection pressure. If some lineages experienced more relaxed or rigorous selection than others in the light of divergence distances, the dN value should reveal a poor linear fit than dS value. Use of nonsynonymous substitutions with lineage-specific selection pressure change could lead to incorrect phylogenetic inference [72].

Sites of C to U RNA-editing in *matR *have been identified experimentally in several angiosperm species [73-76]. Although previous small-scale studies revealed no significant differences in phylogenetic inference between including and excluding RNA-edited sites [34,77], it may be necessary to test for this effect on phylogeny estimation when a large-scale analysis is conducted because these sites are not always conserved among species [76]. In addition, processed paralogs, which may disrupt phylogeny estimation if they are jointly analyzed with vertically transferred DNA [34], can be also detected if a given sequence is relatively free from RNA editing. We used PREP-Mt program [78] with cutoff value of 0.6 for predicting RNA-editing sites in the 174-matR sequences. The resulting data matrix (TreeBASE: M3532) was analyzed and compared with original data matrix to examine effects of RNA editing.

## List of abbreviations

*atpB *– ATP synthase beta subunit, plastid gene

BS – bootstrap

dN – Number of nonsynonymous substitutions per nonsynonymous site

dS – Number of synonymous substitutions per synonymous site

GTR – general time reversible model (a model of DNA sequence evolution)

I + Γ – invariant sites plus gamma distribution

JK – jackknife

*matK *– plastid maturase K gene

*matR *– mitochondrial maturase R gene

ML – maximum likelihood

MP – maximum parsimony

mtDNA – mitochondrial DNA.

*rbcL *– ribulose bisphosphate carboxylase/oxygenase, large subunit, plastid gene

TBR – tree bisection-reconnection branch swapping

## Authors' contributions

XYZ carried out all data analyses, and wrote several sections of this manuscript; MWC, YLQ, DLD, and JHL revised several versions of this manuscript; HZK assisted with analyses and alignment of DNA sequences for phylogenetic analyses; ZDC designed the study, conducted field sampling, generated DNA sequences, and wrote several sections. All authors read and commented on drafts of the manuscript and approved the final manuscript.

## Supplementary Material

Additional file 2 **Best-fit models and parameters for ML analyses**. An MS Excel file gives optimal models and parameters determined in ModelTest for *matR*-174-taxon and several data partitions in 91-taxon four-gene matrix using hierarchical likelihood ratio tests (hLRTs). Click here for file

Additional file 3 **Characteristics of the three codon positions in *matR***. An MS Excel file gives statistics for the three-codon positions in *matR*. Values are based on the one of shortest trees found in 174-taxon matrix of *matR*. Pi, parsimony informative; CI, consistency index; RI, retention index; RC, rescaled consistency index. Click here for file

Additional file 8 **ML tree from the predicted 174-taxon matrix of *matR***. The sites of C to U RNA-editing in *matR *are predicted using PREP-Mt program [75] with cutoff value of 0.6 for predicting RNA-editing sites in the 174-*matR *sequences. The resulting data matrix is analyzed using ML (GTR+Γ model). Click here for file

Additional file 4 **Maximum parsimony and maximum likelihood statistical tests of alternative topologies**. An MS Excel file contains results of statistical tests, the Templeton and Winning-site tests for parsimony topologies, and the approximately unbiased (AU), and Shimodaira-Hasegawa (SH) tests for maximum likelihood topologies. Numbers in parentheses indicate the source of alternative topologies. Asterisks denote significance differences at P < 0.05 in column 4, 5, and 7. Alternative topologies are presented in Additional file 5. Click here for file

Additional file 5 **The alternative topologies used in statistical tests**. An MS Word file contains alternative topology files. Alternative topologies were generated using MacClade v4.06. Click here for file

Additional file 6 **ML tree with branch lengths from the 174-taxon matrix of *matR***. A single tree with branch lengths proportional to the amount of change from the maximum likelihood (ML) analysis of the mitochondrial *matR *gene with 174 taxa using the GTR+Γ model, showing the pattern of long and short branches that occurs repeatedly in flowering plants. Asterisks denote contradictory resolutions between ML tree and MP strict consensus of all shortest trees. Click here for file

Additional file 7 **ML tree with branch lengths from the four-gene matrix**. A single tree with branch lengths proportional to the amount of change from the maximum likelihood (ML) analysis of the four-gene matrix of *matR*, *rbcL*, *atpB *and 18S rDNA using GTR+I+Γ model showing the pattern of long and short branches that occurs repeatedly in flowering plants. Asterisks denote contradictory resolutions between ML tree and MP strict consensus of all shortest trees. Click here for file

Additional file 1 **Taxon sampling for the mitochondrial *matR*and combined data sets**. The MS Excel file provides taxon sampling of the *matR *gene and GenBank accession numbers for *matR *alone and four-gene data sets. Entries in red denote the taxa newly sequenced for *matR *in this study. Click here for file
