# Supplementary figures and images for: Mitochondrial matR sequences help to resolve deep phylogenetic relationships in rosids
Source: BMC Evol Biol. 2007 Nov 10;7:217. doi: 10.1186/1471-2148-7-217 (PMC2222252; doi:10.1186/1471-2148-7-217)

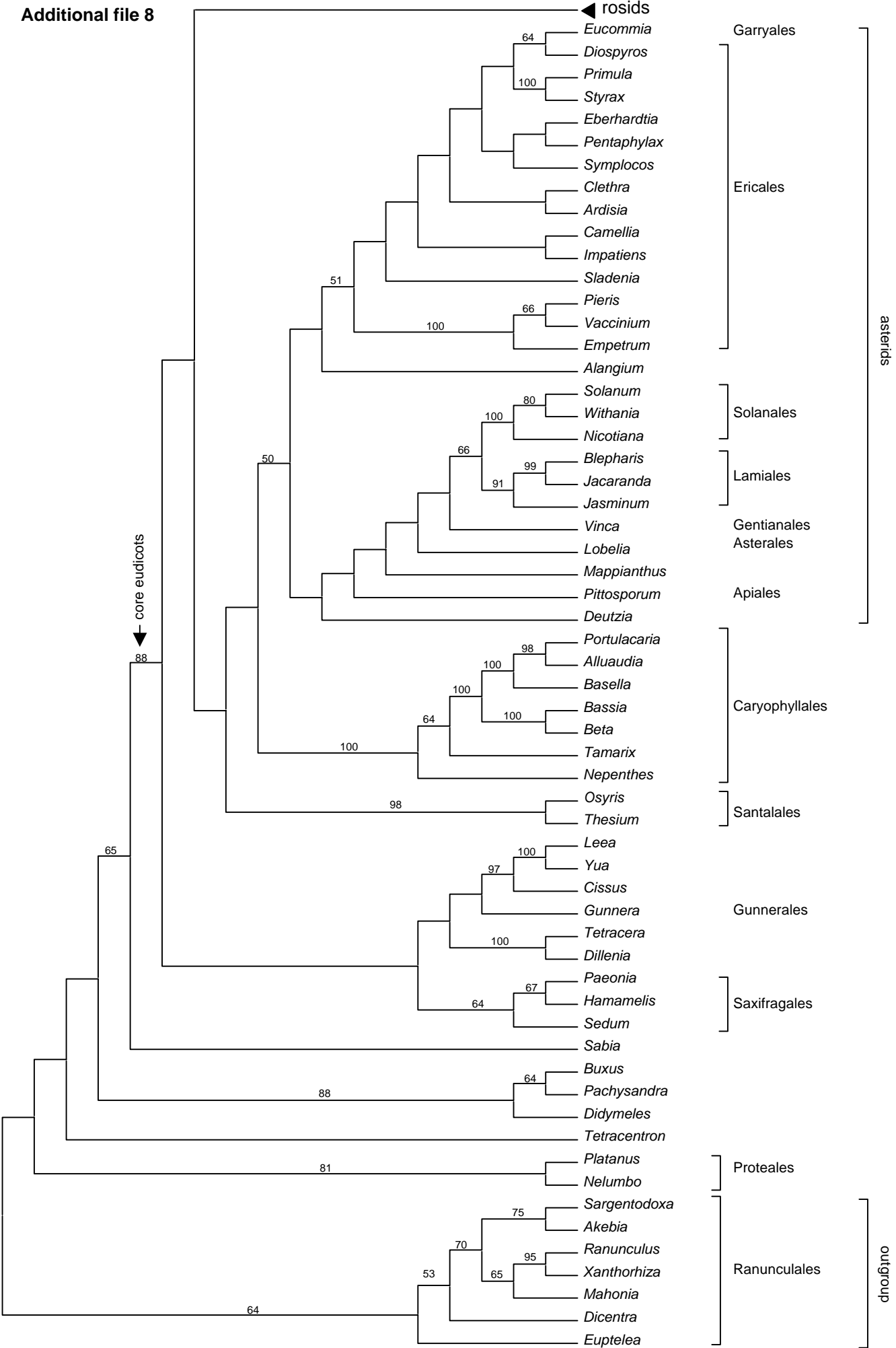

rosids clade

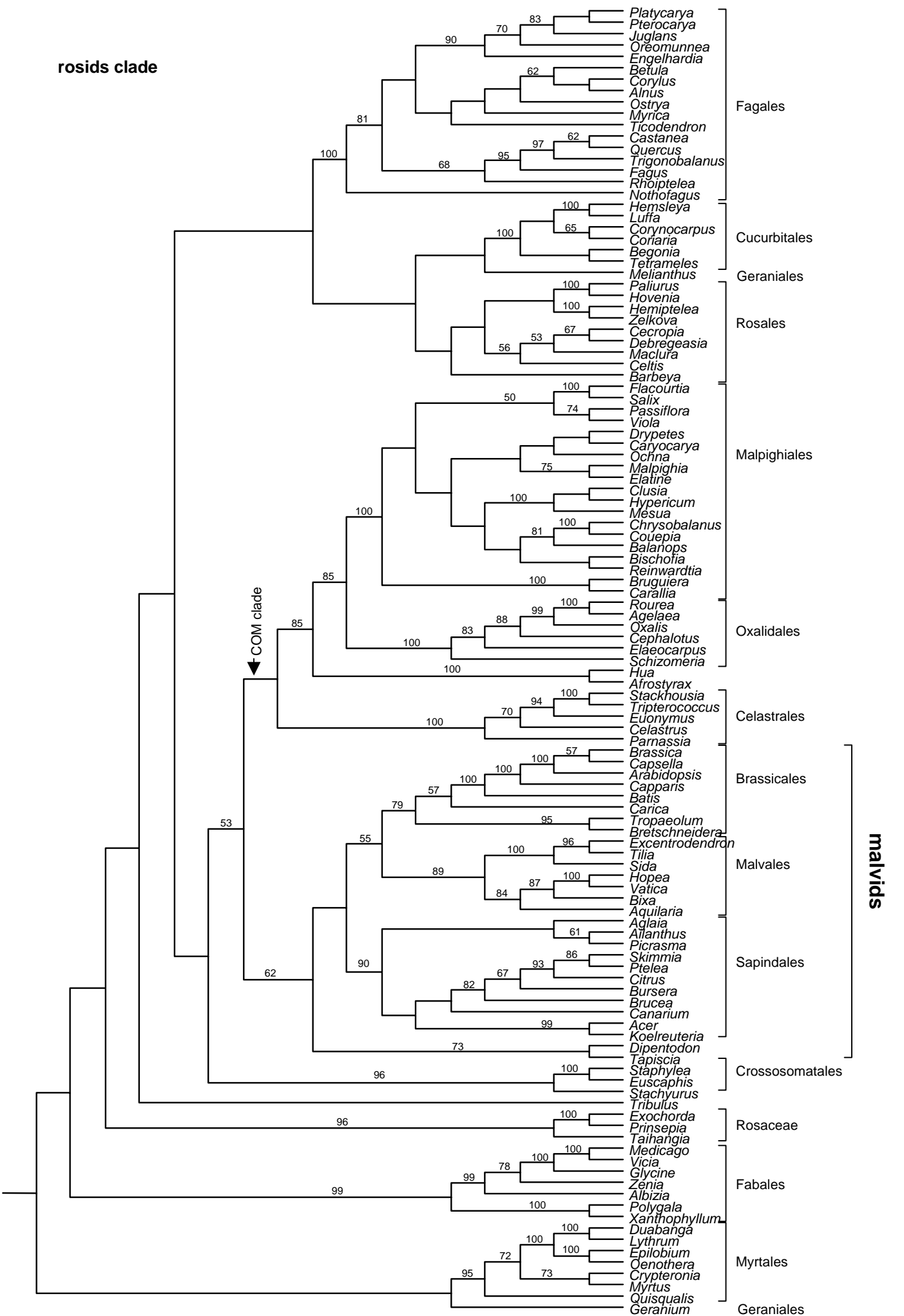

Supplement: Additional file 8 — ML tree from the predicted 174-taxon matrix of matR. The sites of C to U RNA-editing in matR are predicted using PREP-Mt program [75] with cutoff value of 0.6 for predicting RNA-editing sites in the 174-matR sequences. The resulting data matrix is analyzed using ML (GTR+Γ model). [file 1471-2148-7-217-S8.pdf]

Additional file 6

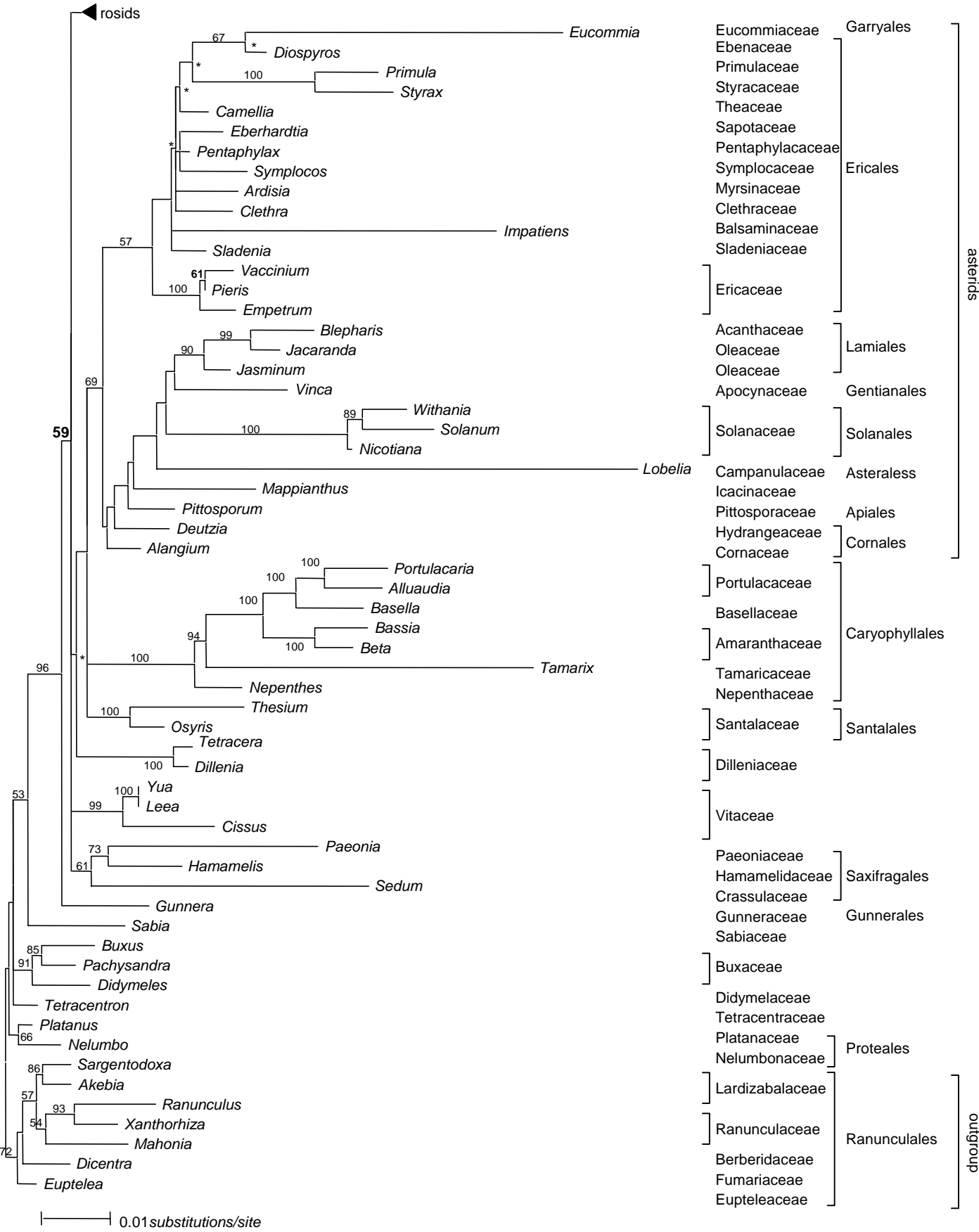

## rosid clade

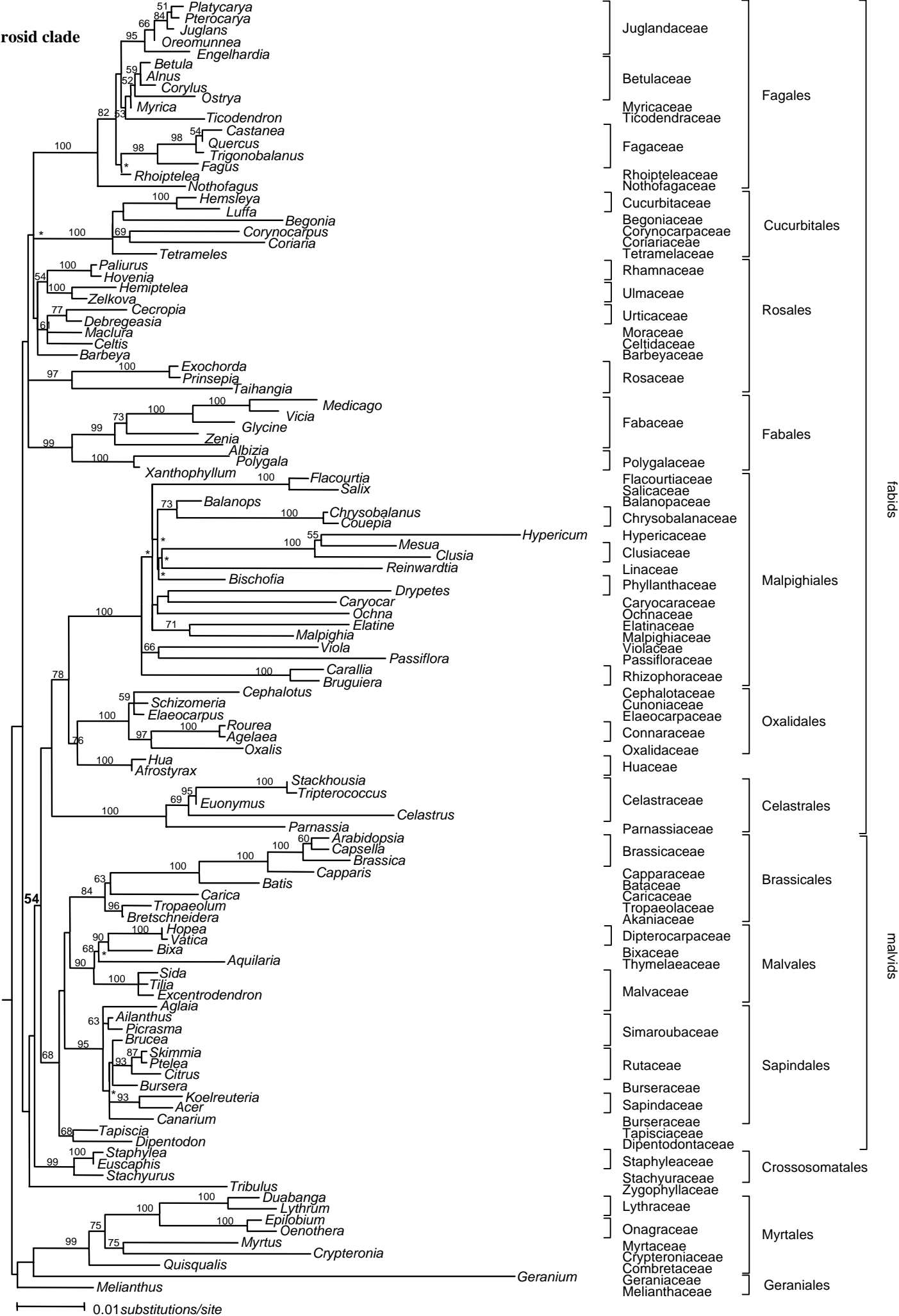

Supplement: Additional file 6 — ML tree with branch lengths from the 174-taxon matrix of matR. A single tree with branch lengths proportional to the amount of change from the maximum likelihood (ML) analysis of the mitochondrial matR gene with 174 taxa using the GTR+Γ model, showing the pattern of long and short branches that occurs repeatedly in flowering plants. Asterisks denote contradictory resolutions between ML tree and MP strict consensus of all shortest trees. [file 1471-2148-7-217-S6.pdf]

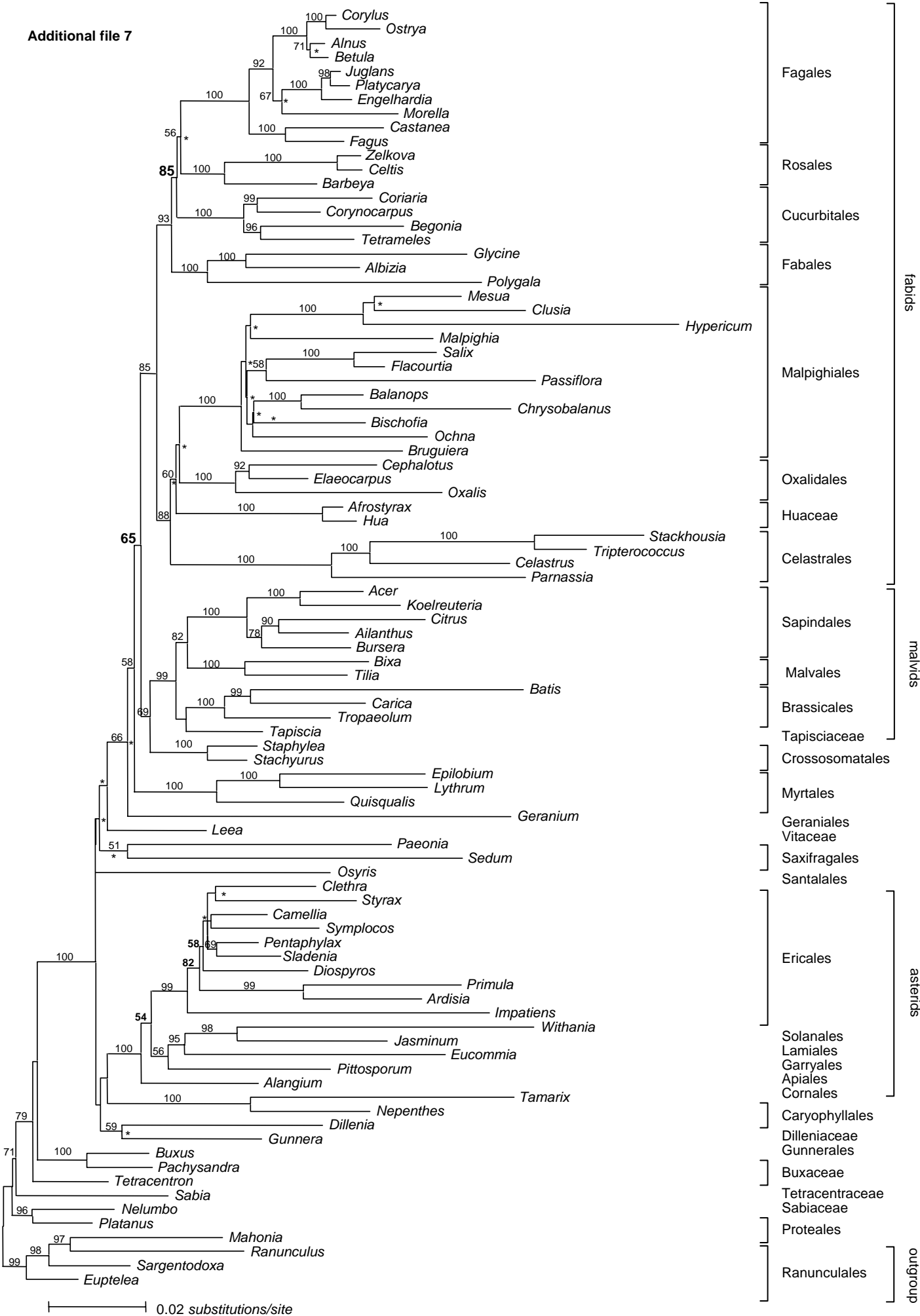

Supplement: Additional file 7 — ML tree with branch lengths from the four-gene matrix. A single tree with branch lengths proportional to the amount of change from the maximum likelihood (ML) analysis of the four-gene matrix of matR, rbcL, atpB and 18S rDNA using GTR+I+Γ model showing the pattern of long and short branches that occurs repeatedly in flowering plants. Asterisks denote contradictory resolutions between ML tree and MP strict consensus of all shortest trees. [file 1471-2148-7-217-S7.pdf]
