# Supplementary material for: Mitochondrial matR sequences help to resolve deep phylogenetic relationships in rosids
Source: BMC Evol Biol. 2007 Nov 10;7:217. doi: 10.1186/1471-2148-7-217 (PMC2222252; doi:10.1186/1471-2148-7-217)
Supplement: Additional file 5 — The alternative topologies used in statistical tests. An MS Word file contains alternative topology files. Alternative topologies were generated using MacClade v4.06. [file 1471-2148-7-217-S5.doc]

**Additional file 4 -**  **The alternative topologies used in statistical tests. The numbers in parentheses indicate the source of alternative topologies**

| Data/method | Hypothesis Tested | Alternative Topologies |
| --- | --- | --- |
| *matR* alone/ parsimony | Huaceae sister to Celastrales (3, 12)  With monophyly of fabids (3,12,15)  Celastrales sister to Oxalidales (15,31)  Celastrales sister to Malpighiales (21) | tree 'hua+cel' = [&U](((((((((((((((((Clethra, ((Sladenia, (Ardisia, (Camellia, Impatiens))), ((Eberhardtia, (Pentaphylax, Symplocos)), (Diospyros, (Styrax, Primula))))), (Empetrum, (Vaccinium, Pieris))), (Pittosporum, (Mappianthus, (Eucommia, ((Vinca, (Jasminum, (Blepharis, Jacaranda))), (Lobelia, (Nicotiana, (Withania, Solanum)))))))), Deutzia), Alangium), (Nepenthes, (Tamarix, ((Basella, (Portulacaria, Alluaudia)), (Bassia, Beta))))), (Osyris, Thesium)), ((Melianthus, (Geranium, (Quisqualis, (((Duabanga, Lythrum), (Epilobium, Oenothera)), (Myrtus, Crypteronia))))), (((((((Hemsleya, Luffa), (Corynocarpus, Coriaria)), (Begonia, Tetrameles)), (Barbeya, (((Paliurus, Hovenia), (Hemiptelea, Zelkova)), (Celtis, (Maclura, (Debregeasia, Cecropia)))))), (((Xanthophyllum, Polygala), (Albizia, (Zenia, (Glycine, (Medicago, Vicia))))), (Nothofagus, (((Engelhardia, (Oreomunnea, (Juglans, (Pterocarya, Platycarya)))), (Ticodendron, (Myrica, (Ostrya, (Betula, (Alnus, Corylus)))))), (Rhoiptelea, (Fagus, (Trigonobalanus, (Quercus, Castanea)))))))), (Taihangia, (Exochorda, Prinsepia))), (Tribulus, (((((Parnassia, (Celastrus, (Euonymus, (Stackhousia, Tripterococcus)))), (Afrostyrax, Hua)), (((((Viola, Passiflora), (Salix, Flacourtia)), ((Bischofia, ((Balanops, (Couepia, Chrysobalanus)), (Carallia, Bruguiera))), ((Ochna, (Caryocar, Drypetes)), (Reinwardtia, (Malpighia, Elatine))))), (Clusia, (Hypericum, Mesua))), ((Oxalis, (Agelaea, Rourea)), (Elaeocarpus, (Schizomeria, Cephalotus))))), ((Dipentodon, Tapiscia), (((Aquilaria, ((Bixa, (Hopea, Vatica)), (Excentrodendron, (Sida, Tilia)))), ((Carica, (Batis, (Capparis, (Brassica, (Capsella, Arabidopsis))))), (Tropaeolum, Bretschneidera))), ((Citrus, (Ptelea, Skimmia)), ((Bursera, Brucea, (Canarium, (Aglaia, (Picrasma, Ailanthus)))), (Koelreuteria, Acer)))))), (Stachyurus, (Staphylea, Euscaphis))))))), (Dillenia, Tetracera)), (Sedum, (Hamamelis, Paeonia))), (Cissus, (Yua, Leea))), Gunnera), Sabia), (Didymeles, (Buxus, Pachysandra))), (Platanus, Nelumbo)), Tetracentron), (Euptelea, (Dicentra, ((Akebia, Sargentodoxa), (Mahonia, (Xanthorhiza, Ranunculus))))));  tree 'fabids' = [&U](((((((((((((((((Clethra, ((Sladenia, (Ardisia, (Camellia, Impatiens))), ((Eberhardtia, (Pentaphylax, Symplocos)), (Diospyros, (Styrax, Primula))))), (Empetrum, (Vaccinium, Pieris))), (Pittosporum, (Mappianthus, (Eucommia, ((Vinca, (Jasminum, (Blepharis, Jacaranda))), (Lobelia, (Nicotiana, (Withania, Solanum)))))))), Deutzia), Alangium), (Nepenthes, (Tamarix, ((Basella, (Portulacaria, Alluaudia)), (Bassia, Beta))))), (Osyris, Thesium)), ((Melianthus, (Geranium, (Quisqualis, (((Duabanga, Lythrum), (Epilobium, Oenothera)), (Myrtus, Crypteronia))))), ((((((((Hemsleya, Luffa), (Corynocarpus, Coriaria)), (Begonia, Tetrameles)), (Barbeya, (((Paliurus, Hovenia), (Hemiptelea, Zelkova)), (Celtis, (Maclura, (Debregeasia, Cecropia)))))), (((Xanthophyllum, Polygala), (Albizia, (Zenia, (Glycine, (Medicago, Vicia))))), (Nothofagus, (((Engelhardia, (Oreomunnea, (Juglans, (Pterocarya, Platycarya)))), (Ticodendron, (Myrica, (Ostrya, (Betula, (Alnus, Corylus)))))), (Rhoiptelea, (Fagus, (Trigonobalanus, (Quercus, Castanea)))))))), (Taihangia, (Exochorda, Prinsepia))), ((Parnassia, (Celastrus, (Euonymus, (Stackhousia, Tripterococcus)))), (((((Viola, Passiflora), (Salix, Flacourtia)), ((Bischofia, ((Balanops, (Couepia, Chrysobalanus)), (Carallia, Bruguiera))), ((Ochna, (Caryocar, Drypetes)), (Reinwardtia, (Malpighia, Elatine))))), (Clusia, (Hypericum, Mesua))), ((Afrostyrax, Hua), ((Oxalis, (Agelaea, Rourea)), (Elaeocarpus, (Schizomeria, Cephalotus))))))), (Tribulus, (((Dipentodon, Tapiscia), (((Aquilaria, ((Bixa, (Hopea, Vatica)), (Excentrodendron, (Sida, Tilia)))), ((Carica, (Batis, (Capparis, (Brassica, (Capsella, Arabidopsis))))), (Tropaeolum, Bretschneidera))), ((Citrus, (Ptelea, Skimmia)), ((Bursera, Brucea, (Canarium, (Aglaia, (Picrasma, Ailanthus)))), (Koelreuteria, Acer))))), (Stachyurus, (Staphylea, Euscaphis))))))), (Dillenia, Tetracera)), (Sedum, (Hamamelis, Paeonia))), (Cissus, (Yua, Leea))), Gunnera), Sabia), (Didymeles, (Buxus, Pachysandra))), (Platanus, Nelumbo)), Tetracentron), (Euptelea, (Dicentra, ((Akebia, Sargentodoxa), (Mahonia, (Xanthorhiza, Ranunculus))))));  tree 'cel+oxa' = [&U](((((((((((((((((Clethra, ((Sladenia, (Ardisia, (Camellia, Impatiens))), ((Eberhardtia, (Pentaphylax, Symplocos)), (Diospyros, (Styrax, Primula))))), (Empetrum, (Vaccinium, Pieris))), (Pittosporum, (Mappianthus, (Eucommia, ((Vinca, (Jasminum, (Blepharis, Jacaranda))), (Lobelia, (Nicotiana, (Withania, Solanum)))))))), Deutzia), Alangium), (Nepenthes, (Tamarix, ((Basella, (Portulacaria, Alluaudia)), (Bassia, Beta))))), (Osyris, Thesium)), ((Melianthus, (Geranium, (Quisqualis, (((Duabanga, Lythrum), (Epilobium, Oenothera)), (Myrtus, Crypteronia))))), (((((((Hemsleya, Luffa), (Corynocarpus, Coriaria)), (Begonia, Tetrameles)), (Barbeya, (((Paliurus, Hovenia), (Hemiptelea, Zelkova)), (Celtis, (Maclura, (Debregeasia, Cecropia)))))), (((Xanthophyllum, Polygala), (Albizia, (Zenia, (Glycine, (Medicago, Vicia))))), (Nothofagus, (((Engelhardia, (Oreomunnea, (Juglans, (Pterocarya, Platycarya)))), (Ticodendron, (Myrica, (Ostrya, (Betula, (Alnus, Corylus)))))), (Rhoiptelea, (Fagus, (Trigonobalanus, (Quercus, Castanea)))))))), (Taihangia, (Exochorda, Prinsepia))), (Tribulus, ((((((Parnassia, (Celastrus, (Euonymus, (Stackhousia, Tripterococcus)))), ((Oxalis, (Agelaea, Rourea)), (Elaeocarpus, (Schizomeria, Cephalotus)))), (Afrostyrax, Hua)), ((((Viola, Passiflora), (Salix, Flacourtia)), ((Bischofia, ((Balanops, (Couepia, Chrysobalanus)), (Carallia, Bruguiera))), ((Ochna, (Caryocar, Drypetes)), (Reinwardtia, (Malpighia, Elatine))))), (Clusia, (Hypericum, Mesua)))), ((Dipentodon, Tapiscia), (((Aquilaria, ((Bixa, (Hopea, Vatica)), (Excentrodendron, (Sida, Tilia)))), ((Carica, (Batis, (Capparis, (Brassica, (Capsella, Arabidopsis))))), (Tropaeolum, Bretschneidera))), ((Citrus, (Ptelea, Skimmia)), ((Bursera, Brucea, (Canarium, (Aglaia, (Picrasma, Ailanthus)))), (Koelreuteria, Acer)))))), (Stachyurus, (Staphylea, Euscaphis))))))), (Dillenia, Tetracera)), (Sedum, (Hamamelis, Paeonia))), (Cissus, (Yua, Leea))), Gunnera), Sabia), (Didymeles, (Buxus, Pachysandra))), (Platanus, Nelumbo)), Tetracentron), (Euptelea, (Dicentra, ((Akebia, Sargentodoxa), (Mahonia, (Xanthorhiza, Ranunculus))))));  tree 'cel+mal' = [&U](((((((((((((((((Clethra, ((Sladenia, (Ardisia, (Camellia, Impatiens))), ((Eberhardtia, (Pentaphylax, Symplocos)), (Diospyros, (Styrax, Primula))))), (Empetrum, (Vaccinium, Pieris))), (Pittosporum, (Mappianthus, (Eucommia, ((Vinca, (Jasminum, (Blepharis, Jacaranda))), (Lobelia, (Nicotiana, (Withania, Solanum)))))))), Deutzia), Alangium), (Nepenthes, (Tamarix, ((Basella, (Portulacaria, Alluaudia)), (Bassia, Beta))))), (Osyris, Thesium)), ((Melianthus, (Geranium, (Quisqualis, (((Duabanga, Lythrum), (Epilobium, Oenothera)), (Myrtus, Crypteronia))))), (((((((Hemsleya, Luffa), (Corynocarpus, Coriaria)), (Begonia, Tetrameles)), (Barbeya, (((Paliurus, Hovenia), (Hemiptelea, Zelkova)), (Celtis, (Maclura, (Debregeasia, Cecropia)))))), (((Xanthophyllum, Polygala), (Albizia, (Zenia, (Glycine, (Medicago, Vicia))))), (Nothofagus, (((Engelhardia, (Oreomunnea, (Juglans, (Pterocarya, Platycarya)))), (Ticodendron, (Myrica, (Ostrya, (Betula, (Alnus, Corylus)))))), (Rhoiptelea, (Fagus, (Trigonobalanus, (Quercus, Castanea)))))))), (Taihangia, (Exochorda, Prinsepia))), (Tribulus, (((((Parnassia, (Celastrus, (Euonymus, (Stackhousia, Tripterococcus)))), ((((Viola, Passiflora), (Salix, Flacourtia)), ((Bischofia, ((Balanops, (Couepia, Chrysobalanus)), (Carallia, Bruguiera))), ((Ochna, (Caryocar, Drypetes)), (Reinwardtia, (Malpighia, Elatine))))), (Clusia, (Hypericum, Mesua)))), ((Afrostyrax, Hua), ((Oxalis, (Agelaea, Rourea)), (Elaeocarpus, (Schizomeria, Cephalotus))))), ((Dipentodon, Tapiscia), (((Aquilaria, ((Bixa, (Hopea, Vatica)), (Excentrodendron, (Sida, Tilia)))), ((Carica, (Batis, (Capparis, (Brassica, (Capsella, Arabidopsis))))), (Tropaeolum, Bretschneidera))), ((Citrus, (Ptelea, Skimmia)), ((Bursera, Brucea, (Canarium, (Aglaia, (Picrasma, Ailanthus)))), (Koelreuteria, Acer)))))), (Stachyurus, (Staphylea, Euscaphis))))))), (Dillenia, Tetracera)), (Sedum, (Hamamelis, Paeonia))), (Cissus, (Yua, Leea))), Gunnera), Sabia), (Didymeles, (Buxus, Pachysandra))), (Platanus, Nelumbo)), Tetracentron), (Euptelea, (Dicentra, ((Akebia, Sargentodoxa), (Mahonia, (Xanthorhiza, Ranunculus)))))); |
| *matR* alone/ likelihood | Huaceae sister to Celastrales (3, 12)  With monophyly of fabids (3,12,15)  Celastrales sister to Oxalidales (15,31)  Celastrales sister to Malpighiales (21) | tree 'hua+cel' = [&U]((((((((((((((((((((((Chrysobalanus, Couepia), Balanops), (Bischofia, (Reinwardtia, (Clusia, (Mesua, Hypericum))))), (Flacourtia, Salix)), ((Ochna, (Drypetes, Caryocar)), (Elatine, Malpighia))), (Passiflora, Viola)), (Bruguiera, Carallia)), ((Elaeocarpus, (Schizomeria, Cephalotus)), (Oxalis, (Rourea, Agelaea)))), ((Hua, Afrostyrax), (Parnassia, (Celastrus, (Euonymus, (Tripterococcus, Stackhousia)))))), ((((Aglaia, (Ailanthus, Picrasma)), (Canarium, ((Acer, Koelreuteria), (Brucea, (Bursera, (Citrus, (Skimmia, Ptelea))))))), (((Bretschneidera, Tropaeolum), (Carica, (Batis, (Capparis, (Brassica, (Arabidopsis, Capsella)))))), ((Aquilaria, (Bixa, (Vatica, Hopea))), (Excentrodendron, (Tilia, Sida))))), (Tapiscia, Dipentodon))), (Stachyurus, (Euscaphis, Staphylea))), Tribulus), ((((Albizia, (Zenia, (Glycine, (Vicia, Medicago)))), (Polygala, Xanthophyllum)), (Taihangia, (Prinsepia, Exochorda))), (((Nothofagus, (((Engelhardia, (Oreomunnea, (Juglans, (Platycarya, Pterocarya)))), (Ticodendron, (Myrica, (Ostrya, (Betula, (Corylus, Alnus)))))), (Rhoiptelea, (Fagus, (Trigonobalanus, (Castanea, Quercus)))))), (Tetrameles, ((Coriaria, Corynocarpus), (Begonia, (Luffa, Hemsleya))))), (Barbeya, ((Celtis, (Maclura, (Cecropia, Debregeasia))), ((Zelkova, Hemiptelea), (Hovenia, Paliurus))))))), (Melianthus, (Geranium, (Quisqualis, ((Crypteronia, Myrtus), ((Oenothera, Epilobium), (Lythrum, Duabanga))))))), (((((Alangium, (Deutzia, (Pittosporum, (Mappianthus, (Lobelia, ((Vinca, (Jasminum, (Jacaranda, Blepharis))), (Nicotiana, (Solanum, Withania)))))))), (((Sladenia, Impatiens), (Clethra, ((Camellia, ((Diospyros, Eucommia), (Primula, Styrax))), (Ardisia, (Symplocos, (Pentaphylax, Eberhardtia)))))), (Empetrum, (Pieris, Vaccinium)))), (Nepenthes, (Tamarix, ((Beta, Bassia), (Basella, (Alluaudia, Portulacaria)))))), (Thesium, Osyris)), (Tetracera, Dillenia))), (Cissus, (Leea, Yua))), (Sedum, (Paeonia, Hamamelis))), Gunnera), Sabia), (Didymeles, (Pachysandra, Buxus))), Tetracentron), ((Euptelea, (Dicentra, ((Sargentodoxa, Akebia), (Mahonia, (Ranunculus, Xanthorhiza))))), (Nelumbo, Platanus)));  tree 'fabids' = [&U] (((((((((((((((((((((Chrysobalanus, Couepia), Balanops), (Bischofia, (Reinwardtia, (Clusia, (Mesua, Hypericum))))), (Flacourtia, Salix)), ((Ochna, (Drypetes, Caryocar)), (Elatine, Malpighia))), (Passiflora, Viola)), (Bruguiera, Carallia)), (((Elaeocarpus, (Schizomeria, Cephalotus)), (Oxalis, (Rourea, Agelaea))), (Hua, Afrostyrax))), (Parnassia, (Celastrus, (Euonymus, (Tripterococcus, Stackhousia))))), (Stachyurus, (Euscaphis, Staphylea))), Tribulus), (((((Aglaia, (Ailanthus, Picrasma)), (Canarium, ((Acer, Koelreuteria), (Brucea, (Bursera, (Citrus, (Skimmia, Ptelea))))))), (((Bretschneidera, Tropaeolum), (Carica, (Batis, (Capparis, (Brassica, (Arabidopsis, Capsella)))))), ((Aquilaria, (Bixa, (Vatica, Hopea))), (Excentrodendron, (Tilia, Sida))))), (Tapiscia, Dipentodon)), ((((Albizia, (Zenia, (Glycine, (Vicia, Medicago)))), (Polygala, Xanthophyllum)), (Taihangia, (Prinsepia, Exochorda))), (((Nothofagus, (((Engelhardia, (Oreomunnea, (Juglans, (Platycarya, Pterocarya)))), (Ticodendron, (Myrica, (Ostrya, (Betula, (Corylus, Alnus)))))), (Rhoiptelea, (Fagus, (Trigonobalanus, (Castanea, Quercus)))))), (Tetrameles, ((Coriaria, Corynocarpus), (Begonia, (Luffa, Hemsleya))))), (Barbeya, ((Celtis, (Maclura, (Cecropia, Debregeasia))), ((Zelkova, Hemiptelea), (Hovenia, Paliurus)))))))), (Melianthus, (Geranium, (Quisqualis, ((Crypteronia, Myrtus), ((Oenothera, Epilobium), (Lythrum, Duabanga))))))), (((((Alangium, (Deutzia, (Pittosporum, (Mappianthus, (Lobelia, ((Vinca, (Jasminum, (Jacaranda, Blepharis))), (Nicotiana, (Solanum, Withania)))))))), (((Sladenia, Impatiens), (Clethra, ((Camellia, ((Diospyros, Eucommia), (Primula, Styrax))), (Ardisia, (Symplocos, (Pentaphylax, Eberhardtia)))))), (Empetrum, (Pieris, Vaccinium)))), (Nepenthes, (Tamarix, ((Beta, Bassia), (Basella, (Alluaudia, Portulacaria)))))), (Thesium, Osyris)), (Tetracera, Dillenia))), (Cissus, (Leea, Yua))), (Sedum, (Paeonia, Hamamelis))), Gunnera), Sabia), (Didymeles, (Pachysandra, Buxus))), Tetracentron), ((Euptelea, (Dicentra, ((Sargentodoxa, Akebia), (Mahonia, (Ranunculus, Xanthorhiza))))), (Nelumbo, Platanus)));  tree'cel+oxa' = [&U]((((((((((((((((((((((Chrysobalanus, Couepia), Balanops), (Bischofia, (Reinwardtia, (Clusia, (Mesua, Hypericum))))), (Flacourtia, Salix)), ((Ochna, (Drypetes, Caryocar)), (Elatine, Malpighia))), (Passiflora, Viola)), (Bruguiera, Carallia)), (Hua, Afrostyrax)), (((Elaeocarpus, (Schizomeria, Cephalotus)), (Oxalis, (Rourea, Agelaea))), (Parnassia, (Celastrus, (Euonymus, (Tripterococcus, Stackhousia)))))), ((((Aglaia, (Ailanthus, Picrasma)), (Canarium, ((Acer, Koelreuteria), (Brucea, (Bursera, (Citrus, (Skimmia, Ptelea))))))), (((Bretschneidera, Tropaeolum), (Carica, (Batis, (Capparis, (Brassica, (Arabidopsis, Capsella)))))), ((Aquilaria, (Bixa, (Vatica, Hopea))), (Excentrodendron, (Tilia, Sida))))), (Tapiscia, Dipentodon))), (Stachyurus, (Euscaphis, Staphylea))), Tribulus), ((((Albizia, (Zenia, (Glycine, (Vicia, Medicago)))), (Polygala, Xanthophyllum)), (Taihangia, (Prinsepia, Exochorda))), (((Nothofagus, (((Engelhardia, (Oreomunnea, (Juglans, (Platycarya, Pterocarya)))), (Ticodendron, (Myrica, (Ostrya, (Betula, (Corylus, Alnus)))))), (Rhoiptelea, (Fagus, (Trigonobalanus, (Castanea, Quercus)))))), (Tetrameles, ((Coriaria, Corynocarpus), (Begonia, (Luffa, Hemsleya))))), (Barbeya, ((Celtis, (Maclura, (Cecropia, Debregeasia))), ((Zelkova, Hemiptelea), (Hovenia, Paliurus))))))), (Melianthus, (Geranium, (Quisqualis, ((Crypteronia, Myrtus), ((Oenothera, Epilobium), (Lythrum, Duabanga))))))), (((((Alangium, (Deutzia, (Pittosporum, (Mappianthus, (Lobelia, ((Vinca, (Jasminum, (Jacaranda, Blepharis))), (Nicotiana, (Solanum, Withania)))))))), (((Sladenia, Impatiens), (Clethra, ((Camellia, ((Diospyros, Eucommia), (Primula, Styrax))), (Ardisia, (Symplocos, (Pentaphylax, Eberhardtia)))))), (Empetrum, (Pieris, Vaccinium)))), (Nepenthes, (Tamarix, ((Beta, Bassia), (Basella, (Alluaudia, Portulacaria)))))), (Thesium, Osyris)), (Tetracera, Dillenia))), (Cissus, (Leea, Yua))), (Sedum, (Paeonia, Hamamelis))), Gunnera), Sabia), (Didymeles, (Pachysandra, Buxus))), Tetracentron), ((Euptelea, (Dicentra, ((Sargentodoxa, Akebia), (Mahonia, (Ranunculus, Xanthorhiza))))), (Nelumbo, Platanus)));  tree 'cel+mal' = [&U]((((((((((((((((((((((Chrysobalanus, Couepia), Balanops), (Bischofia, (Reinwardtia, (Clusia, (Mesua, Hypericum))))), (Flacourtia, Salix)), ((Ochna, (Drypetes, Caryocar)), (Elatine, Malpighia))), (Passiflora, Viola)), (Bruguiera, Carallia)), (Parnassia, (Celastrus, (Euonymus, (Tripterococcus, Stackhousia))))), (((Elaeocarpus, (Schizomeria, Cephalotus)), (Oxalis, (Rourea, Agelaea))), (Hua, Afrostyrax))), ((((Aglaia, (Ailanthus, Picrasma)), (Canarium, ((Acer, Koelreuteria), (Brucea, (Bursera, (Citrus, (Skimmia, Ptelea))))))), (((Bretschneidera, Tropaeolum), (Carica, (Batis, (Capparis, (Brassica, (Arabidopsis, Capsella)))))), ((Aquilaria, (Bixa, (Vatica, Hopea))), (Excentrodendron, (Tilia, Sida))))), (Tapiscia, Dipentodon))), (Stachyurus, (Euscaphis, Staphylea))), Tribulus), ((((Albizia, (Zenia, (Glycine, (Vicia, Medicago)))), (Polygala, Xanthophyllum)), (Taihangia, (Prinsepia, Exochorda))), (((Nothofagus, (((Engelhardia, (Oreomunnea, (Juglans, (Platycarya, Pterocarya)))), (Ticodendron, (Myrica, (Ostrya, (Betula, (Corylus, Alnus)))))), (Rhoiptelea, (Fagus, (Trigonobalanus, (Castanea, Quercus)))))), (Tetrameles, ((Coriaria, Corynocarpus), (Begonia, (Luffa, Hemsleya))))), (Barbeya, ((Celtis, (Maclura, (Cecropia, Debregeasia))), ((Zelkova, Hemiptelea), (Hovenia, Paliurus))))))), (Melianthus, (Geranium, (Quisqualis, ((Crypteronia, Myrtus), ((Oenothera, Epilobium), (Lythrum, Duabanga))))))), (((((Alangium, (Deutzia, (Pittosporum, (Mappianthus, (Lobelia, ((Vinca, (Jasminum, (Jacaranda, Blepharis))), (Nicotiana, (Solanum, Withania)))))))), (((Sladenia, Impatiens), (Clethra, ((Camellia, ((Diospyros, Eucommia), (Primula, Styrax))), (Ardisia, (Symplocos, (Pentaphylax, Eberhardtia)))))), (Empetrum, (Pieris, Vaccinium)))), (Nepenthes, (Tamarix, ((Beta, Bassia), (Basella, (Alluaudia, Portulacaria)))))), (Thesium, Osyris)), (Tetracera, Dillenia))), (Cissus, (Leea, Yua))), (Sedum, (Paeonia, Hamamelis))), Gunnera), Sabia), (Didymeles, (Pachysandra, Buxus))), Tetracentron), ((Euptelea, (Dicentra, ((Sargentodoxa, Akebia), (Mahonia, (Ranunculus, Xanthorhiza))))), (Nelumbo, Platanus))); |
| Combined data /parsimony | Malvales sister to Brassicales (12, 15) | tree 'mal+bra' = [&U](((((((((((((((Celastrus, (Stackhousia, Tripterococcus)), Parnassia), (Hua, Afrostyrax)), (((((Malpighia, Bruguiera), (Mesua, (Clusia, Hypericum))), (Passiflora, (Flacourtia, Salix))), (Ochna, Bischofia)), (Balanops, Chrysobalanus))), (Oxalis, (Cephalotus, Elaeocarpus))), (((((Corynocarpus, Coriaria), (Tetrameles, Begonia)), ((Morella, ((Betula, (Alnus, (Ostrya, Corylus))), (Engelhardia, (Platycarya, Juglans)))), (Fagus, Castanea))), (Barbeya, (Celtis, Zelkova))), (Polygala, (Albizia, Glycine)))), ((Stachyurus, Staphylea), (((Tapiscia, (Tropaeolum, (Carica, Batis))), (Bixa, Tilia)), ((Bursera, (Ailanthus, Citrus)), (Acer, Koelreuteria))))), (Quisqualis, (Epilobium, Lythrum))), Leea), (((Gunnera, (Geranium, Sedum)), ((Alangium, ((Pittosporum, (Eucommia, (Withania, Jasminum))), (Impatiens, ((Clethra, (((Camellia, Symplocos), (Pentaphylax, Sladenia)), (Diospyros, Styrax))), (Ardisia, Primula))))), (Dillenia, (Nepenthes, Tamarix)))), (Paeonia, Osyris))), (Pachysandra, Buxus)), Tetracentron), Sabia), (Platanus, Nelumbo)), (Euptelea, (Sargentodoxa, (Ranunculus, Mahonia)))); |
| Combined data/likelihood | Huaceae sister to Celastrales (3,12)  Malvales sister to Brassicales (12, 15) | tree 'hua+cel' = [&U] ((((((((((((((((((Tripterococcus, Stackhousia), Celastrus), Parnassia), (Afrostyrax, Hua)), ((Bruguiera, ((Malpighia, (Hypericum, (Clusia, Mesua))), ((Passiflora, (Salix, Flacourtia)), (Ochna, (Bischofia, (Chrysobalanus, Balanops)))))), (Oxalis, (Elaeocarpus, Cephalotus)))), ((((Barbeya, (Zelkova, Celtis)), ((((Corylus, Ostrya), (Alnus, Betula)), (Morella, (Engelhardia, (Juglans, Platycarya)))), (Castanea, Fagus))), ((Coriaria, Corynocarpus), (Begonia, Tetrameles))), (Polygala, (Glycine, Albizia)))), ((((Tilia, Bixa), ((Bursera, (Citrus, Ailanthus)), (Koelreuteria, Acer))), (Tapiscia, (Tropaeolum, (Batis, Carica)))), (Staphylea, Stachyurus))), (Quisqualis, (Lythrum, Epilobium))), Geranium), Leea), (Sedum, Paeonia)), Osyris), ((Dillenia, Gunnera), ((Alangium, ((Impatiens, ((Diospyros, ((Clethra, Styrax), ((Camellia, Symplocos), (Pentaphylax, Sladenia)))), (Primula, Ardisia))), (Pittosporum, (Eucommia, (Jasminum, Withania))))), (Tamarix, Nepenthes)))), (Buxus, Pachysandra)), Tetracentron), Sabia), (Nelumbo, Platanus)), (Euptelea, (Sargentodoxa, (Mahonia, Ranunculus))));  tree 'mal+bra' = [&U] (((((((((((((((((Tripterococcus, Stackhousia), Celastrus), Parnassia), (((Bruguiera, ((Malpighia, (Hypericum, (Clusia, Mesua))), ((Passiflora, (Salix, Flacourtia)), (Ochna, (Bischofia, (Chrysobalanus, Balanops)))))), (Oxalis, (Elaeocarpus, Cephalotus))), (Afrostyrax, Hua))), ((((Barbeya, (Zelkova, Celtis)), ((((Corylus, Ostrya), (Alnus, Betula)), (Morella, (Engelhardia, (Juglans, Platycarya)))), (Castanea, Fagus))), ((Coriaria, Corynocarpus), (Begonia, Tetrameles))), (Polygala, (Glycine, Albizia)))), ((((Bursera, (Citrus, Ailanthus)), (Koelreuteria, Acer)), ((Tilia, Bixa), (Tapiscia, (Tropaeolum, (Batis, Carica))))), (Staphylea, Stachyurus))), (Quisqualis, (Lythrum, Epilobium))), Geranium), Leea), (Sedum, Paeonia)), Osyris), ((Dillenia, Gunnera), ((Alangium, ((Impatiens, ((Diospyros, ((Clethra, Styrax), ((Camellia, Symplocos), (Pentaphylax, Sladenia)))), (Primula, Ardisia))), (Pittosporum, (Eucommia, (Jasminum, Withania))))), (Tamarix, Nepenthes)))), (Buxus, Pachysandra)), Tetracentron), Sabia), (Nelumbo, Platanus)), (Euptelea, (Sargentodoxa, (Mahonia, Ranunculus)))); |
